# Interventions aiming to reduce time to antibiotics (TTA) in patients with fever and neutropenia during chemotherapy for cancer (FN), a systematic review

**DOI:** 10.1007/s00520-019-05056-w

**Published:** 2019-09-05

**Authors:** Christa Koenig, Christine Schneider, Jessica E. Morgan, Roland A. Ammann, Lillian Sung, Bob Phillips

**Affiliations:** 1 Division of Pediatric Hematology/Oncology, Department of Pediatrics, Inselspital, Bern University Hospital, University of Bern, Freiburgstrasse 15, CH-3010 Bern, Switzerland; 2 Centre for Reviews and Dissemination, University of York, York, UK; 3 Leeds Children’s Hospital, Leeds, UK; 4 The Hospital for Sick Children, University of Toronto, Toronto, Ontario Canada

**Keywords:** Oncology, Cancer, Fever, Neutropenia, Time to antibiotics, Quality improvement projects, Systematic review

## Abstract

**Purpose:**

Multiple interventions have been developed aiming to reduce time to antibiotics (TTA) in patients with fever and neutropenia (FN) following chemotherapy for cancer. We evaluated their effect to reduce TTA and their impact on important clinical outcomes in a systematic review.

**Methods:**

The search covered seven databases. Biases and quality of studies were assessed with the Risk of Bias in Non-randomized Studies of Interventions (ROBINS-I) tool. Interventions could be implemented in any setting and performed by any person included in the FN management. Absolute change of TTA was the primary outcome. Registration: PROSPERO (CRD42018092948).

**Results:**

Six thousand two hundred ninety-six titles and abstracts were screened, 177 studies were retrieved and 30 studies were included. Risk of bias was moderate to serious in 28 studies and low in two studies. All but one study reported a reduction of TTA after the intervention. Various types of interventions were implemented; they most commonly aimed at professionals. Most of the studies made more than one single intervention.

**Conclusion:**

This review may help centers to identify their specific sources of delay and barriers to change and to define what intervention may be the best to apply. This review supports the assertion that TTA can be considered a measure of quality of care, emphasizes the importance of education and training, and describes the very different interventions which have effectively reduced TTA.

**Electronic supplementary material:**

The online version of this article (10.1007/s00520-019-05056-w) contains supplementary material, which is available to authorized users.

## Background

In patients with cancer, fever in chemotherapy-induced severe neutropenia (FN) is the most frequent potentially lethal complication of chemotherapy for cancer [1]. When absolute neutrophil count drops below 0.5 × 10^9^/L the risk of life-threatening bacterial infection increases [2]. Prompt empirical therapy with broad-spectrum antibiotics is standard of care and lethality is below 1% in pediatric patients [3, 4] and approximately 10% in adult patients [5], but still FN remains the leading cause of emergency hospitalization. Time to antibiotics (TTA) usually refers to the amount of time passed from arrival at the hospital to administration of antibiotics, and despite inconsistent evidence about the association of TTA and clinical outcomes, guidelines [6–8] and experts insist that timely and appropriate antibiotic administration is essential for adequate patient care. TTA < 60 min is even used as a measure of quality of care [9]. Presuming the beneficial effect on patient-important outcomes, several groups have attempted to reduce TTA in patients with FN by implementing specific interventions in emergency departments (ED) and oncology wards. These interventions have never been summarized, so this systematic review aimed to identify and synthesize information on interventions performed, their effect to reduce TTA, and the potential use of these approaches.

## Methods

The protocol for this review was registered on PROSPERO (CRD42018092948) prior to commencing the work and has been published [10]. Simultaneously with this systematic review, we collected information about the association between TTA and clinical outcomes in patients with FN under chemotherapy for cancer, published separately [11]. This section is an adapted version of the methods reported there.

Electronic searches of MEDLINE, MEDLINE In-Process & Other Non-Indexed Citations, EMBASE, CINAHL, CDSR, CENTRAL, and LILACS were performed on May 9th, 2018. The search was updated on April 5th, 2019. The search strategy included the Medical Subject Heading terms and text words to identify fever and neutropenia and the intervention of treatment with antibiotics. Antibiotics were additionally searched by groups and names of antibiotic drugs (e.g., penicillin, beta-lactams, quinolones).

In EMBASE search, “time” was added as a required search factor to narrow the results. Studies from 1997 onward were eligible, no language restrictions were applied. Pilot searching took place before the actual search and found all five previous identified studies [12–16]. The full search strategies are provided with the protocol publication [10]. Manual searches of references and forward citation searching of included articles was conducted. Authors of relevant studies and experts within the field were contacted to seek further studies.

### Study selection

Inclusion and exclusion criteria were defined a priori. Studies investigating any intervention or combination of interventions attempting to reduce TTA in adult or pediatric patients with cancer, or after hematopoietic stem cell transplantation, and FN were included. Interventions could be implemented in inpatient or outpatient settings, performed by any person included in the FN management. All kind of studies, except case reports and those presented only as abstract or posters, were eligible.

#### Outcomes

Absolute change of TTA was the primary outcome. Secondary outcomes were TTA measurements other than absolute, safety, and treatment adequacy. Safety was defined as death, admission to intensive care unit (ICU), and/or severe sepsis (including septic shock); treatment adequacy was defined as relapse of primary infection, persistence of fever, and/or recurrence of fever without a new infection. Additional clinical outcomes as microbiologically defined infections, days of fever, length of hospital stay (LOS), modification of antibiotics, new infections, and composite outcomes that each individual study selected were recorded.

#### Exclusion criteria

Studies were excluded if (1) they were not specific to cancer or did not report on this subgroup separately (mixed populations were permitted if > 50% population were diagnosed with cancer/hematopoietic stem cell transplantation); (2) they did not report TTA; (3) they did not have data of an accurate comparator group, defined as cared for in the same way, in the same setting, and with the same treatment regimens, except of the intervention studied. The comparison group could be of the same cohort and could be observed simultaneously or successively.

#### Screening

One reviewer (CK) screened the title and abstract of all studies for inclusion. A second reviewer (CS) independently screened 60% of the titles and abstracts. The kappa statistic for agreement was calculated and showed good agreement between reviewers (*k* = 0.91, 95% confidence interval (CI) 0.87 to 0.94). Full text was obtained for all potential articles of interest. All full texts were assessed for eligibility by two reviewers (CK and CS; *k* = 0.79, 95% CI 0.69 to 0.89). Fourteen studies were referred to a third reviewer (RSP), where 11 were excluded.

### Data extraction and risk of bias assessment

Data extraction and risk of bias assessment was done by one reviewer (CK) and independently checked by a second (RAA). Discrepancies were resolved by consensus. Intervention characteristics were collected according to the Cochrane Effective Practice and Organization of Care Review Group (EPOC) data collection checklist [17]. Risk of bias was assessed using the Risk of Bias in Non-randomized Studies of Interventions (ROBINS-I) tool [18] at the level of the individual study. All articles were included in the review irrespective of the risk of bias.

### Statistical methods

Due to heterogeneity within the implemented interventions, study sites, and participants, meta-analysis was not undertaken and a narrative synthesis was performed. To visually display the results of the primary outcome, reduction of TTA, a forest plot was drawn including all studies for which mean and standard deviation (SD) were reported or could be estimated. In studies only reporting median, interquartile range (IQR) or 95% confidence intervals of the mean, and SD were estimated assuming a normal distribution ($\mathrm{median}=\mathrm{mean};\mathrm{SD}=\mathrm{IQR}/1.35;\mathrm{SD}=95\%\mathrm{CI}/3.92\times \sqrt{n}$) [19].

## Results

### Overview

**Fig. 1:**
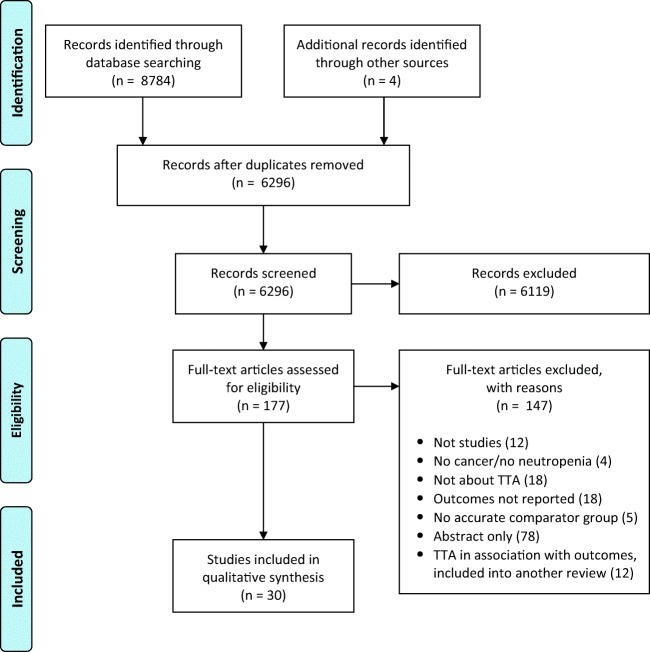
PRISMA flow diagram of identification and selection of eligible studies

Titles and abstracts from 6296 studies were assessed and 177 full-text articles retrieved. A flow diagram of the study selection is provided in Fig. 1. Thirty studies were included, thirteen in adult [12, 20–31], and seventeen in pediatric patients [16, 32–47], including a total of 1891 and 6820 FN episodes, respectively. Two-third of the studies were undertaken in the USA (*n* = 20; 67%). There were four multicenter studies (included number of centers, 2 to 4) and the vast majority of studies were undertaken in academic hospitals (*n* = 25; 83%). No randomized or quasi-randomized trials were identified by the searches. Before and after studies were the most commonly used design (*n* = 29; 97%), in which TTA was evaluated at baseline and after the implementation of an intervention. Various studies collected TTA at multiple time points, but none of them performed an interrupted time series analysis. The remaining study was a retrospective cohort study [26].

Characteristics of included studies are given in Table 1. Most of the interventions were implemented at EDs (26; 87%). Fever was defined within a temperature range of ≥ 38.0 to ≥ 38.5 °C. Seventeen studies defined neutropenia as an absolute neutrophil count (ANC) < 0.5 × 10^9^/L and/or < 1.0 × 10^9^/L expected to decrease. Four studies defined neutropenia as ANC < 1.0 × 10^9^/L. Other definitions were leucocyte count ≤ 4.0 × 10^9^/L or < 1.0 × 10^9^/L, ANC < 0.2 × 10^9^/L, or < 0.58 × 10^9^/L and expected to decrease. TTA was measured from triage or arrival at the hospital to first dose of antibiotics in the majority of studies (*n* = 26; 87%). One study with adult patients started measurement of time at fever detection [30], one study defined TTA as time from initial provider evaluation to intravenous antibiotic administration, [44], and the third study started measurement at ICU admission [32]. In one study, a definition was lacking [36]. The definitions used per study are displayed in Online Resource 1, Table 1.

**Table 1:** Characteristics of included studies. Italicized data has been calculated by combining data from two arms

Study	Year	Country	Single or	Data collection	Intervention design defined by each study authors	Setting			FN episodes	Assessed
	pub	(language)	multisite			ED	Outpatient unit	Inpatient unit	(patients)	risk of bias
Adult patients										
Baltic et al. [20]	2002	USA (E)	Single site	Retrospective	Quality improvement project	Yes	Yes	Yes	*11*	Serious
Best et al. [21]	2011	USA (E)	Single site	Retrospective	Practice change process	Yes	No	Yes	*53 (53)*	Moderate
Dang et al. [22]	2018	USA (E)	Single site	Prospective	Define, measure, analyze, improve, control (DMAIC) process improvement project	Yes	No	No	*216 (216)*	Serious
Hawley et al. [23]	2011	USA (E)	Single site	Retrospective	Multidisciplinary Team Project	Yes	Yes	No	42 (42)	Serious
Kapil et al. [12]	2016	Canada (E)	Single site	Retrospective	Fever advisory cards implementation	Yes	No	No	*308 (308)*	Moderate
Keng et al. [24]	2015	USA (E)	Single site	Retrospective and prospective	Implementation of a FN Pathway	Yes	No	No	497(386)	Low
Ko et al. [25]	2015	China (E)	Single site	Retrospective	Implementation of protocol	Yes	No	No	69 (69)	Moderate
Lim et al. [26]	2012	Canada (E)	Multisite	Retrospective	Electronic clinical practice guideline implementation	Yes	No	No	*201 (201)*	Serious
Lim et al. [27]	2013	China (E)	Single site	Retrospective	Changes to improve performance	Yes	Yes	No	*62 (62)*	Moderate
Meisenberg et al. [28]	2015	USA (E)	Single site	Retrospective	Performance improvement project	Yes	Yes	Yes	*69 (69)*	Moderate
Salter et al. [29]	2005	Australia (E)	Single site	Retrospective	Pathway implementation	Yes	No	No	*35*	Serious
Van Vliet et al. [30]	2011	Netherlands (E)	Single site	Prospective	Strategies for improvement	No	Yes	No	*187 (167)*	Moderate
Wells et al. [31]	2015	UK (E)	Single site	Retrospective and prospective	Service developments	Yes	Yes	Yes	*141*	Serious
Pediatric patients										
Amado et al. [32]	2011	Brazil (E)	Single site	Retrospective	Quality improvement project	No	No	ICU	45 (45)	Moderate
Benner et al. [33]	2016	USA (E)	Single site	Retrospective	Quality improvement project	Yes	No	No	253 (111)	Moderate
Cash et al. [34]	2014	USA (E)	Single site	Retrospective	Implementation of standardized process	Yes	No	No	130	Serious
Cohen et al. [35]	2016	USA (E)	Single site	Retrospective and prospective	Protocol initiation	Yes	No	No	*253 (253)*	Moderate
Corey et al. [36]	2008	USA (E)	Single site	Retrospective and prospective	Plan-do-study-act cycle	NA	NA	NA	61	Serious
Dobrasz et al. [37]	2013	USA (E)	Multisite	Retrospective	Evidence-based practice change, quality initiative	Yes	No	No	2768	Serious
Emerson et al. [38]	2018	USA (E)	Single site	Retrospective and prospective	Plan-do-study-act cycles	Yes	No	No	80 (80)	Serious
Lamble et al. [39]	2015	USA (E)	Single site	Retrospective	Clinical care pathway implementation	Yes	No	No	*476(250)*	Moderate
Lukes et al. [40]	2019	USA (E)	Single site	Retrospective	Quality improvement project	Yes	No	No	101 (101)	Moderate
Monroe et al. [41]	2018	USA (E)	Single site	Retrospective and prospective	Quality improvement project	Yes	No	No	NA	Serious
Pakakasama et al. [42]	2011	Thailand (E)	Single site	Retrospective	Establishment of clinical practice guidelines	Yes	No	No	*308 (162)*	Serious
Salstrom et al. [16]	2015	USA (E)	Single site	Retrospective and prospective	Quality improvement project	Yes	No	Yes	*116 (116)*	Moderate
Spencer et al. [43]	2017	USA (E)	Multisite	Retrospective	Quality improvement project	Yes	No	No	1032 (1032)	Moderate
Vanderway et al. [44]	2017	USA (E)	Single site	Retrospective and prospective	Quality improvement project	No	Yes	No	*25 (25)*	Moderate
Vedi et al. [45]	2015	Australia (E)	Multisite	Retrospective	Algorithm-based approach	Yes	No	No	*89 (89)*	Moderate
Volpe et al. [46]	2012	USA (E)	Single site	Retrospective	Quality improvement project, plan-do-study act cycle	Yes	No	No	*365 (365)*	Low
Yoshida et al. [47]	2018	USA (E)	Single site	Prospective	2-phase quality improvement project	Yes	No	No	718 (327)	Moderate

*E*, English; *ED*, emergency department; *FN*, fever and neutropenia; *ICU*, intensive care unit; *NA*, not available; *pub*, published

### Risk of bias

Study quality and risk of bias assessment identified a moderate or serious risk for bias in all but two of the included studies (Table 1, for full assessment: Online Resource 1, Table 2). Potential confounders for TTA were set at FN diagnosis, localization of presentation (ED versus oncology ward versus oncology outpatient unit), high patient volumes, presence of central line, and knowledge of staff about an ongoing study (Hawthorne effect) [32]. Additionally, risk status of patients, initial illness severity, time of presentation, and administration route of antibiotics were identified as possible but measurable confounders in almost all studies.

**Table 2:** Intervention and time to antibiotics reduction in the included studies

Study	Type of intervention				Staff education	Checklist/guidelines	Follow-up	TTA before intervention (mean)	TTA after intervention (mean)	Absolute TTA reduction	% TTA reduction	% with TTA ≤ 60 min before/after
	Professional	Organizational										
		Provider	Patient	Structural								
Baltic et al. [20]	Yes	Yes	Yes	Yes	Yes	Yes	No	188 min	64 min	124 min	66%	NA
Best et al. [21] (1) Best et al. [21] (2)	Yes	No	No	Yes	Yes	Yes	No	188 min	115 min	73 min	38%	NA
	Yes	No	No	Yes	Yes	Yes	No	228 min	163 min	65 min	29%	
Dang et al. [22]	Yes	Yes	Yes	Yes	Yes	Yes	No	100 min	27 min	73 min	73%	31%/95.5%
Hawley et al. [23] (1)	Yes	Yes	Yes	Yes	Yes	Yes	No	138 min	91.6 min	46.4 min	34%	NA
Hawley et al. [23] (2)	Yes	Yes	Yes	Yes	Yes	Yes	No	70 min	52.6 min	17.4 min	25%	NA
Kapil et al. [12]	Yes	No	No	No	No	No	No	244 min	195 min	49 min	20%	NA
Keng et al. [24]	Yes	No	No	Yes	Yes	Yes	No	235 min*	81 min*	154 min	66%	1%/32%
Ko et al. [25]	No	No	No	Yes	NA	Yes	No	300 min	47 min	253 min	84%	0 to 86%
Lim et al. [26]	Yes	No	No	No	Yes	Yes	No	4.9 h*	3.9 h*	1 h	20%	NA
Lim et al. [27]	Yes	Yes	No	Yes	Yes	Yes	No	261 min *	95 min*	166 min	64%	NA
Meisenberg et al. [28]	Yes	No	Yes	Yes	Yes	Yes	No	252 min*	117 min*	135 min	54%	NA
Salter et al. [29]	No	Yes	No	Yes	No	Yes	No	3.8 h*	3.45 h*	0.35 h	9%	NA
Van Vliet et al. [30]	No	Yes	No	Yes	No	No	Yes	75.1 min	32.0 min	43.1 min	57%	NA
Wells et al. [31]	Yes	Yes	No	Yes	Yes	Yes	No	NA	NA	NA	NA	31%/79%
Amado et al. [32]	No	No	No	Yes	No	No	No	164 min	55 min	109 min	66%	0%/52%
Benner et al. [33]	Yes	Yes	Yes	Yes	Yes	Yes	Yes	207 min*	88.5 min*	118.5 min	57%	0.9%/67%
Cash et al. [34]	Yes	Yes	No	Yes	Yes	Yes	No	154 min*	95 min*	59 min	38%	2%/3%
Cohen et al. [35]	Yes	Yes	Yes	Yes	Yes	Yes	Yes	96.9 min	69.5 min	27.4 min	28%	35%/51.4%
Corey et al. [36]	Yes	Yes	No	Yes	Yes	No	Yes	TTA only graphically reported		NA	NA	NA
Dobrasz et al. [37] (1) Dobrasz et al. [37] (2)	Yes	Yes	No	Yes	Yes	Yes	Yes	89 min	44 min	45 min	51%	NA
	Yes	Yes	No	Yes	Yes	Yes	Yes	110 min	61 min	49 min	45%	NA
Emerson et al. [38]	Yes	Yes	Yes	Yes	Yes	Yes	No	116 min	55 min	61 min	53%	NA
Lamble et al. [39]	Yes	No	No	Yes	Yes	Yes	Yes	115 min*	60 min*	55 min	48%	12%/46%
Lukes et al. [40]	Yes	Yes	No	Yes	Yes	No	Yes	108 min*	47 min*	61 min	56%	17%/83%
Monroe et al. [41]	Yes	Yes	Yes	Yes	Yes	Yes	No	NA	NA	NA	NA	30%/80.4%
Pakakasama et al. [42]	Yes	Yes	No	Yes	Yes	Yes	No	180 min*	75 min*	105 min	58%	NA
Salstrom et al. [16]	Yes	No	Yes	Yes	Yes	No	Yes	164 min	45.2 min	119 min	73%	19 to 74%
Spencer et al. [43] (1) Spencer et al. [43] (2) Spencer et al. [43] (3)	Yes	Yes	Yes	Yes	NA	Yes	Yes	118.5 min*	57 min*	61.5 min	52%	NA
	Yes	Yes	Yes	Yes	NA	Yes	Yes	163 min*	97.5 min*	65.6 min	40%	NA
	Yes	Yes	Yes	Yes	NA	Yes	Yes	188 min*	111.5 min*	76.5 min	41%	NA
Vanderway et al. [44]	Yes	Yes	No	Yes	Yes	Yes	No	79.6 min	41.2 min	38.4 min	48%	NA
Vedi et al. [45] (1) Vedi et al. [45] (2)	Yes	Yes	No	Yes	Yes	Yes	Yes	148 min	76 min	72 min	49%	0 to 35%
	Yes	Yes	No	Yes	Yes	Yes	Yes	221 min	65 min	156 min	71%	NA
Volpe et al. [46]	Yes	Yes	No	Yes	Yes	No	No	99 min	49 min	50 min	51%	50%/88.5%
Yoshida et al. [47]	Yes	Yes	No	Yes	Yes	Yes	No	83 min	65 min	18 min	22%	47%/69%

*ED*, emergency department; *NA*, not available; *ICU*, intensive care unit; *TTA*, time to antibiotics; *Median

### Interventions

Various types of interventions were implemented, most of the studies made more than one single intervention. Among the thirty studies, the most common group of intervention targeted professionals. They consisted of distribution of FN-Alert cards to patients, skills training, education for staff, and educational updates or feedbacks. Twenty-two studies implemented guidelines, algorithms, or checklists for FN treatment. The collected interventions are summarized in Online Resource 1, Table 3. No study used regulatory or financial interventions.

**Table 3:** Sources of delays (number of studies reporting) in adult and pediatric patients

Adult patients	Pediatric patients
Staff related	Staff related
• Lack of awareness of potential risk/knowledge about FN (5) • FN patients not recognized (3) • Difficulties in obtaining central venous access (3) • Long waiting time for phlebotomist to set up the intravenous line (1) • Long turn-around time from setting of the prescription by pharmacist to drug dispensing (1) • Antibiotics not scheduled as urgency by pharmacist (1) • Lack of staff (1) • Long waiting time for initial physician assessment (3) • Delay related to waiting for a second medical review (1) • Physician trainees involved in care (1) • Communication issues (1)	• Lack of awareness of potential risk/knowledge about FN (2) • FN patients not recognized (1) • Difficulties in obtaining central venous access due to lack of training/technical difficulties (4) • No physician available (for examination or order for antibiotics) (4) • Lack of expertise, fear of treating oncology patients (3) • Communication with specialist/staff (3)
Patient related	Patient related
• Lack of knowledge of patients (1)	• Difficulties in obtaining central venous access due to inadequate topical analgesia (3) • Difficulties in obtaining central venous access due to parents requesting specific nurses/other expectations (3)
Procedure related	Procedure related
• Lack of a triage system (1) • Unavailability of laboratory results (4) • Missing FN protocols (1) • Absence of order set (2) • Large number of patients, multiple concomitant admissions (1) • Antibiotics not available at emergency (1) • Delayed administration of antibiotics due to structural issues (3) (after transfer to inpatient unit, only on next drug round) • Lack of access to important patient information (1) • Day of the week (1)	• Unawareness of patient arrival (1) • Difficulties in obtaining central venous access due to lack of equipment (1) • Unavailability of laboratory results (6) • Antibiotics not available at emergency (5) • Two separate policies for BMT and non-BMT patients (1) • ED crowding/Competing unwell patients (1) • No exam/infusion room available, room not prepared (2) • Guidelines not accessible (1) • Patient information cannot be entered into the computer system until the patient’s arrival on the unit (1) • Lack of access to important patient information (1)

*BMT*, bone marrow transplant; *ED*, emergency department; *FN*, fever and neutropenia

The studies were indexed to setting, type of intervention, education of staff, implementation of guidelines, algorithms or checklists, and whether they had a follow-up or not (Table 2). Online Resource 1, Table 3 gives an overview of the applied interventions. The number of intervention events varied from 1 to 7, and duration of intervention was from one single intervention point up to 3 years. Interventions were provided and delivered by local physicians, nurses, pharmacist, laboratory staff, and employees from administration and hospital bed control. One study was supported by hospital quality improvement experts [46] and one by members of the Information Technology department [40]. Unit of allocation and analysis were always the individual patients, and the purpose of recommendation was always appropriate management of these patients.

Targeted behaviors were diagnosis (*n* = 13), test ordering (*n* = 6), procedures (*n* = 19), prescribing (*n* = 12), general management of a problem (*n* = 19), patient education/advice (*n* = 11), and communication between professionals (*n* = 8). To address them, various formats were used like interpersonal, paper, visual, computer, paging system, and phones.

Several articles identified sources of delays and barriers to improve TTA; these are presented separately between adult and pediatric studies in Table 4.

**Table 4:** Barriers to change (number of studies reporting) in adult and pediatric patients

Adult patients	Pediatric patients
Staff related	Staff related
• Disempowerment of clinicians diagnosing and caring for the patient (1) • Lack of understanding, ignorance or stubbornness, concerns with lack of autonomy (physician) (2) • Lack of order set compliance, low guideline usage (2) • Lack of communication between professionals (1) • Lack of ongoing education (1) • ED overcrowding, (not enough staff) (1)	• Persisting mind-set to confirm neutropenia before antibiotics (1) • False sense of security: “just another FN patient” (1) • Lack of knowledge due to rotating residents/medical students (1) • Education difficulties due to part-time and rotate shifts (2) • Lack of guideline compliance (3) • Forgetting the availability of standard dose of antibiotics (1) • Overwhelming workload, priorities for other patients (2)
Patient related	Patient related
• Fever alert card unsuitable for some patients (to big, neglect the replacement of a full card) (1) • Inadequate consultations/intolerance of patients (1)	• Patient/parents preferences of staff for central venous access (2)
Procedure related	Procedure related
• Lack of communication of order-set-availability (1)	• Institution not used to standardized processes (1) • State regulation (protocol cannot be initiated before an attending physician assumes care for a patient) (1) • Already close to target before intervention (1)

*ED*, emergency department; *FN*, fever and neutropenia

### Reduction of TTA

All 28 studies that compared TTA before and after an intervention reported a reduction in TTA after the intervention (Table 2). Only one of these studies [12], performed in adults, reports a statistically non-significant result, even when TTA declined from a mean of 244 to 195 min (*p* = 0.09). This study was judged at moderate risk for bias. One study displayed TTA only graphically [36], but equally shows a reduction of TTA. The remaining retrospective cohort study [26] compared TTA in four different hospitals while the intervention was only implemented in one. Likewise, this study showed a significant shorter TTA in the intervention hospital (3.9 versus 4.9 h, *p* = 0.02).

**Fig. 2:**
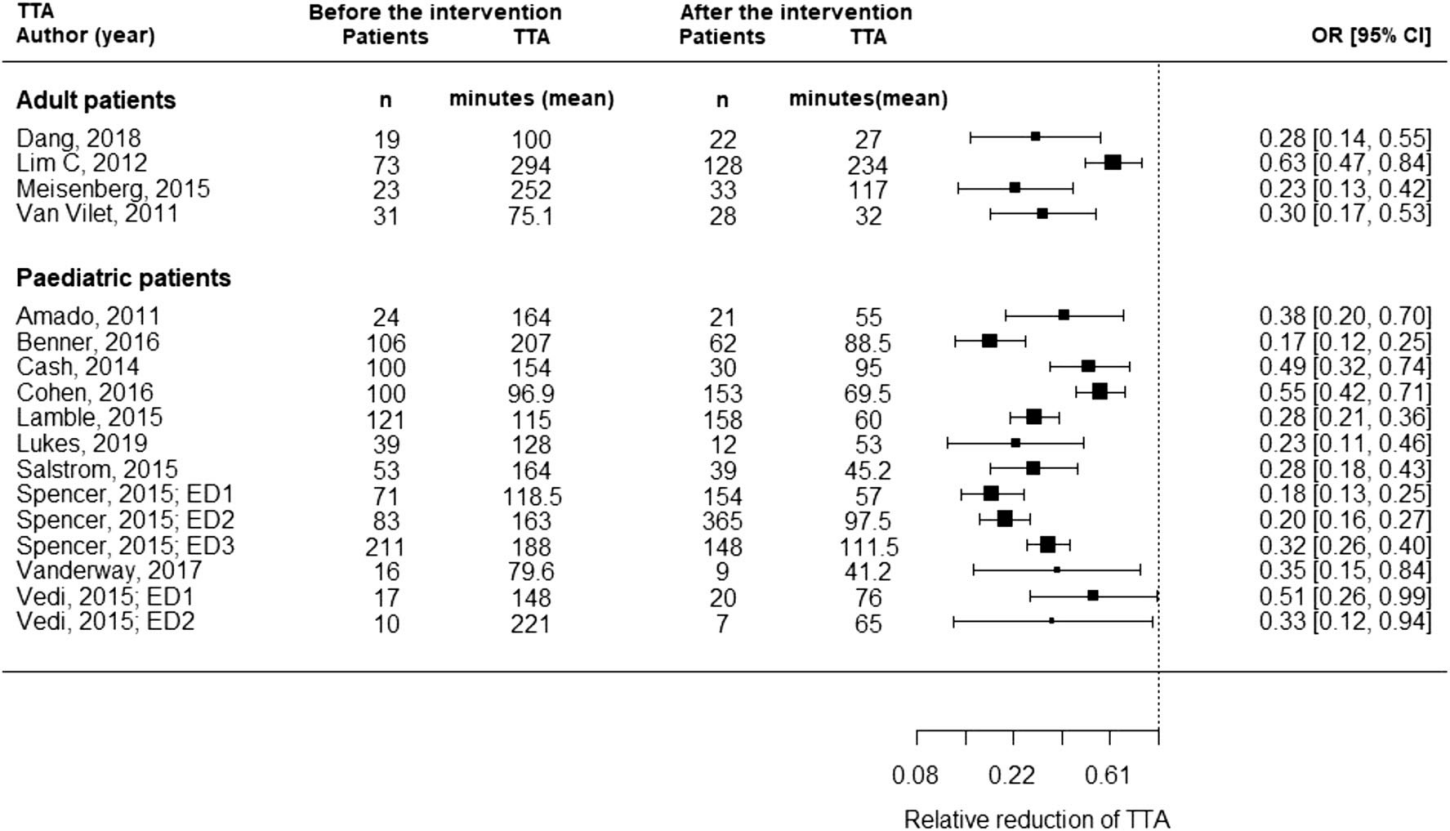
Relative reduction in TTA of studies reporting mean and standard deviation or when those parameters could be estimated

TTA is reported as continuous variable in all but two studies [31, 41]. These specific studies only report an increase in percentage of patients treated within 60 min, as it was reported by several other studies, in addition to continuous TTA (Table 2). The relative reduction of TTA is displayed in Fig. 2 for studies reporting mean and SD or when those parameters could be estimated.

### Clinical outcomes

#### Safety

Most studies were underpowered to address safety (mortality, ICU admission, or occurrence of severe sepsis). Number of deaths was reported by eleven studies [16, 24, 25, 31, 33, 35, 36, 39, 42, 43, 46], with a median mortality of 0% (maximum 39%) before and median mortality of 0% (maximum 6%) after the intervention. No significant differences of mortality before and after intervention were detected in seven of these 11 studies. One study only reported the overall number of deaths [43]. One study [25] found an increase in mortality from 0 (0/19 episodes) to 6% (3/50 episodes; *p* < 0.05), but these are equally low numbers of deaths and no significant difference was found when death was included into a composite outcome together with serious medical complications (1/19 versus 7/50; *p* = 0.45). A decrease in mortality was shown in two studies [31, 42]. For adult patients, Wells et al. [31] report a high mortality of 39% (14 of 36 episodes) before and a mortality of 0% (0 of 79 episodes) after their intervention. Their intervention increased the percentage of patients treated within 60 min from 14 to 79%, but no absolute times are reported. For pediatric patients, Pakakasama et al. [42] reports a significant reduction in mortality from 6.5 (9 of 138 episodes) before to 0% (0 of 170 episodes; *p* = 0.001) after the implementation of guidelines. In their study, ICU admission and septic shock were as well significantly reduced after the implementation, 9.4 to 2.9% (*p* = 0.016) and 10.9 to 3.5% (*p* = 0.011), respectively.

Numbers of ICU admissions were reported by eight studies [24, 25, 33, 35, 36, 39, 42, 46], with a median of 5% (maximum 9%) before and a median of 1.3% (maximum 7%) after the intervention. Only one study [16] found a difference in need for ICU admission before and after the intervention (34% versus 12.8%; *p* < 0.05). This study describes extending the study period when the results were not significant, without describing the number or nature of the interim analyses. Sepsis was additionally analyzed by one adult study [25], where almost all patients before (84%) and after the intervention (90%) were diagnosed with sepsis.

#### Treatment adequacy

No study reported relapses of primary infection, persistence of fever for more than 5 days, or recurrence of fever without a new infection.

#### Additional outcomes

Numbers of patients with an identified source of infection/bacteremia were comparable within the investigated groups before and after the intervention in all studies that analyzed these [16, 24, 27, 39, 42]. Likewise none of three studies with data about duration of fever found a significant difference within the groups [16, 25, 32].

For length of hospital stay (LOS), five studies [24, 25, 27, 29, 33] did not find a significant difference before and after the intervention. In one of those studies [24], LOS was reduced after an intervention when compared with a historical cohort in a multivariable analysis that adjusted for age, disease type, MASCC risk index, prophylactic antibiotics, central line, and ANC, but not in univariate analysis. Only the pediatric study of Pakakasama et al. [42], who described improved safety, showed that LOS decreased significantly after the intervention. Median LOS was 5 days (range, 1–30 days) in the 170 episodes of the intervention group, whereas it was 7 days (range, 1–170 days) in the 138 episodes of the control group (*p* = 0.001). In one study [21], LOS was shorter before (mean 11.33 days; *n* = 30) than after the intervention (mean 17.43 days; *n* = 23). The authors explain this finding by low number of patients and outliers due to unequal groups. Modification of antibiotics and new infections were not reported by any study.

### Subgroup analyses

Only the planned subgroup analyses between pediatric and adult patients were possible to be undertaken. The main finding was different sources of delays in these two groups (Table 3). Regardless of these different barriers, the durations and reductions were broadly similar: TTA before an intervention varied between an average (median or mean) of 70–300 min in adult studies and 79.6–221 min in pediatric studies. After an intervention, adult studies showed TTAs between 27 and 234 min and pediatric studies between 41.2–111.5 min. Reduction of the average TTA was between 17.8–253 min (or 9–84%) in adult and 18–156 min (or 22–73%) in pediatric studies.

## Discussion

TTA can be effectively reduced by very different interventions in a wide range of practice settings in both pediatric and adult patients with fever and neutropenia during chemotherapy for cancer. Most of the identified interventions were aimed at modifying the behavior of professionals and implemented at EDs; implementation of guidelines or a checklist were the most often used strategies. Although it is reasonable to assume that publication bias strongly influences this result if only effective interventions are reported, this systematic review helps to identify possible sources of delays and summarizes different strategies to address them.

Guidelines or checklists are useful to address patient-, procedure-, and staff-related factors at once and they were used by 77% of the included studies. Whereas patient- and procedure-related factors may be more difficult to address otherwise, staff-related factors can be resolved by regular education and training. Unsurprisingly, staff-related factors were also a common reason for delays of TTA and professional interventions were very often used. A systematic review evaluating effective knowledge translation strategies in cancer [48] found that the most promising interventions were professional ones, like educational outreach, audits, and feedbacks. In line with these results, our systematic review supports the fact that education and training remain core elements for a successful reduction in TTA. One study showed a reduction in median TTA about 1 h, only by informing staff about already existing guidelines [26]. Unfortunately staff-related issues were also the most often identified barriers to change (Table 4).

Published interventions without professional approaches were all organizational: standing orders allowing nurses to administer antibiotics before calling a physician [29, 30], making antibiotics rapidly available [32], and implementation of a treatment protocol without emphasizing staff education [25]. Among them was the study with the largest TTA reduction [25], with a reduction of mean TTA by 253 min, keeping in mind that this study also reports the longest TTA before the organizational change (mean 300 min).

Presentation at the ED has been identified as a reason for longer TTA [15, 49] and more frequent adverse events [14]. This matches our finding that 26 (87%) of the identified studies were undertaken in EDs. High workload due to high patient volumes and lack of training in care of oncology patients may explain this. Three included studies [26, 34, 47] mentioned ED overcrowding and lack of staff as a barrier to changes; these are factors difficult to address by physicians but should be acknowledged by institutions, where identified.

A study from Canada [50] identified age > 60 years and lack of caregiver as a risk factor for delayed TTA. In our review, only three studies identified patient-related factors as barriers to change; this may reflect the largely positive input of patients or lack of specific research into these issues.

There were several challenges to summarizing the primary data sources. Through differences in the definitions of key study variables, it was not possible to identify specific interventions that are more likely to be effective than others. The studies were undertaken in different countries and their results must be interpreted in the context of different healthcare provisions. Additionally, the interventions were uneven with respect to type of intervention, number and duration of interventions, what person delivered the intervention, target of behavior, and format used. Because often a number of changes were instituted simultaneously, it is not possible to determine the impact of any single change.

Almost all studies were before and after studies. The identified Hawthorne effect (knowledge of staff about an ongoing study) may have an important influence and therefore follow-up assessments after the intervention should be undertaken to see whether the improvements are sustainable. Outcomes, such as TTA, may change over time for reasons unrelated to the implemented strategy. If repeated observations before and after an intervention are available, the more robust interrupted time series analysis should be conducted [51]. Assessment of clinical outcomes was limited due to low numbers. Additionally, inclusion of patients at different risk for medical complications creates a triage bias, i.e., faster treatment of patients with worse clinical condition [11] and may therefore mask an effect of shorter TTA in the included studies.

The key strength of this manuscript lies in its thorough application of systematic review methodology. It thus provides the most complete summary of interventions aiming to reduce TTA in patients with FN during chemotherapy for cancer. Additionally, it provides a clearly arranged list of sources of delays and barriers to change TTA, and the undertaken risk of bias assessment helps to judge the validity of the results in TTA reduction.

## Conclusion

TTA can be effectively reduced by very different interventions; however, the direct impact of a shorter TTA on clinical outcomes could not be determined. Education and training were identified as core elements to successfully reduce TTA and remain essential to improve quality of care. Some centers already defined TTA ≤ 60 min as a valid measure of quality of care [9, 24], and although the clinical implication of a shorter TTA is not yet clear [11], our results support the assertion that TTA can be considered a measure of quality of care. This systematic review can be used by care teams as a checklist to identify sources of delays and to evaluate what may be the most important and effective intervention to implement in their specific center to reduce TTA.

## Electronic supplementary material

ESM 1 (PDF 623 kb)
