# Supplementary material for: Interventions aiming to reduce time to antibiotics (TTA) in patients with fever and neutropenia during chemotherapy for cancer (FN), a systematic review
Source: Support Care Cancer. 2019 Sep 5;28(5):2369–80. doi: 10.1007/s00520-019-05056-w (PMC7083808; doi:10.1007/s00520-019-05056-w)
Supplement: Supplementary file 1 — (PDF 623 kb) [file 520_2019_5056_MOESM1_ESM.pdf]

Online Resource 1: C Koenig*, C Schneider, JE Morgan, RA Ammann, L Sung, B Phillips. Interventions aiming to reduce time to antibiotics (TTA) in patients with fever and neutropenia during chemotherapy for cancer (FN), a systematic review. Supportive Care in Cancer

Study (year pub)	Fever definition	Neutropenia definition	Time to antibiotics definition
**Adult patients**			
Baltic et al. [20] (2002)	No information	ANC <1.0x10^{9}/L or leucocyte count ≤4.0x10^{9}/L	Arrival at hospital or diagnosis of FN to initiation of antibiotic therapy.
Best et al. [21] (2011)	≥38.3°C or ≥38.0°C over one hour	ANC <0.5x10^{9}/L	Admission to the ED or oncology unit and the initiation of antibiotic therapy
Dang et al. [22] (2018)	No information	No information	Emergency Severity Index acuity entered in electronic medical record to antibiotic administration
Hawley et al. [23] (2011)	≥38.1°C	ANC <0.5x10^{9}/L	Door to antibiotic
Kapil et al. [12] (2016)	No information	No information	Time of ED admission to time of medication dispensation
Keng et al. [24] (2015)	≥ 38.3°C or >38.0°C for greater than 1 hour	ANC <0.5x10^{9}/L	Time from registration until antibiotic administration
Ko et al. [25] (2015)	>38.3°C	ANC <1.0x10^{9}/L	Door to antibiotic
Lim et al. [26] (2012)	>38.0°C	With blood cells <1.0x10^{9}/L or ANC <0.5x10^{9}/L	Interval from triage to initial administration of antibiotics
Lim et al. [27] (2013)	≥38.3°C or >38.0°C for at least 1 hour	ANC <0.5x10^{9}/L or <1.0x10^{9}/L with a predicted decrease to <0.5x10^{9}/L	Arrival to ED or outpatient clinic to administration of first dose of antibiotics
Meisenberg et al. [28] (2015)	≥38.3°C or ≥38.0°C sustained for 1h	ANC <0.5x10^{9}/L or counts expected to decrease to that level over the ensuing 48h	From presentation to the ED to administration of the antibiotics
Salter et al. [29] (2005)	No information	No information	Door to antibiotic time
Van Vliet et al. [30] (2011)	≥38.5°C	Considered to be immunocompromised because of their underlying disease or its treatment with chemotherapy	Detection of fever to administration of the first does of medication
Wells et al. [31] (2015)	>38.0°C or other signs or symptoms consistent with clinically significant sepsis	ANC <1.0x10^{9}/l	Presentation to hospital (door time) to administration of antibiotics (needle time)
Paediatric patients			
Amado et al. [32] (2011)	No information	ANC <0.5x10^{9}/L or undergoing GCS-F administration for neutropenia	Hour of ICU admission to time of antibiotic delivery
Benner et al. [33] (2016)	≥38.3°C or ≥38.0°C on 2 occasions	ANC ≤0.5x10^{9}/L or ≤1.0x10^{9}/L with active chemotherapy and falling counts compared with prior data	Arrival to antibiotic administration
Cash et al. [34] (2014)	≥38.3°C	No information	From triage to first dose of antibiotics
Cohen et al. [35] (2016)	≥38.3°C or 38.0 – 38.2°C twice within a 12 hour period	ANC <0.5x10^{9}/L	From triage check-in to infusion of initial antibiotic dose
Corey et al. [36] (2018)	No information	No information	No information
Dobrasz et al. [37] (2013)	>38°C for longer than 1 hour or ≥38.3°C	ANC <0.5x10^{9}/L	Greet to antibiotics
Emerson et al. [38] (2018)	No information	No information	From patient arrival to broad-spectrum antibiotic time
Lamble et al. [39] (2015)	>38.3°C or two episodes >38.0°C measured 1 hour apart	ANC <0.5x10^{9}/L	Arrival time to antibiotics

*Corresponding author: C Koenig, Division of Pediatric Hematology/Oncology, Department of Pediatrics, Inselspital, Bern University Hospital, University of Bern, Bern, Switzerland; and Centre of Reviews and Dissemination, University of York, UK, christa.koenig@insel.ch

Lukes et al. [40] (2019)	No information	ANC <0.5x10^9/L within the last 48h	ED triage to antibiotic administration
Monroe et al. [41] (2018)	≥ 38.3°C or 2 times between 38.0 and 38.2°C within a 12 hours	ANC <0.5x10^9/L	Time from triage to antibiotic administration
Pakakasama et al. [42] (2011)	No information	ANC <0.5x10^9/L and ANC 0.5 – 1.0x10^9/L	Door-to-antibiotic time
Salstrom et al. [16] (2015)	>38.3°C or multiple fevers >38.0°C over a 24h period	ANC <0.5x10^9/L or ANC <0.58x10^9/L and expected to fall	Check-in time or time of first fever for in clinic patients, to start of intravenous antibiotic administration
Spencer et al. [43] (2017)	No information	No information	ED arrival to ED nurse documentation of first antibiotic administration
Vanderway et al. [44] (2017)	No information	ANC ≤0.5x10^9/L	Initial provider evaluation to intravenous antibiotic administration
Vedi et al. [45] (2015)	>38°C	ANC <1.0x10^9/L	Presentation to triage to time of administration of the first antibiotic
Volpe et al. [46] (2012)	≥38.3°C or ≥38.0°C for more than 2 hours in 24 hours	ANC <0.5x10^9/L	Initial arrival at ED to antibiotic administration
Yoshida et al. [47] (2018)	≥38.3°C or >38.0°C for more than 8 hours	ANC <0.2x10^9/L or dropping ANC or presuming/functional neutropenia as determined by the hemato-/oncologist	Arrival to the ED to first antibiotic

Online Resource 1, Table 1: Definitions used by the included studies. *ANC*, absolute neutrophil count; *ED*, emergency department; *FN*, fever and neutropenia; *GCS-F*, granulocyte colony-stimulation factor; *ICU*, intensive care unit; *pub*, published

*Corresponding author: C Koenig, Division of Pediatric Hematology/Oncology, Department of Pediatrics, Inselspital, Bern University Hospital, University of Bern, Bern, Switzerland; and Centre of Reviews and Dissemination, University of York, UK, christa.koenig@insel.ch

Author and citation	Year pub	Baseline confounding	Selection of participants	Classification of intervention	Deviation from intended intervention	Missing data	Measurement of outcomes	Selection of reported results	Overall Risk of bias
Adult patients									
Baltic et al. [20]	2002	serious	low	low	NI	NI	moderate	low	**serious**
Best et al. [21]	2011	moderate	low	low	low	low	moderate	moderate	**moderate**
Dang et al. [22]	2018	serious	low	moderate	low	low	moderate	moderate	**serious**
Hawley et al. [23]	2011	serious	NI	NI	NI	NI	low	low	**serious**
Kapil et al. [12]	2016	moderate	low	low	moderate	low	moderate	low	**moderate**
Keng et al. [24]	2015	low	low	low	low	NI	Low-moderate	low	**low**
Ko et al. [25]	2015	moderate	low	Low	low	NI	moderate	low	**moderate**
Lim et al. [26]	2012	serious	low	moderate	moderate	moderate	moderate	low	**serious**
Lim et al. [27]	2013	moderate	low	low	low	low	moderate	low	**moderate**
Meisenberg et al. [28]	2015	moderate	low	low	low	low	moderate	low	**moderate**
Salter et al. [29]	2005	serious	low	low	low	serious	moderate	low	**serious**
Van Vliet et al. [30]	2011	moderate	low	low	low	serious	moderate	low	**moderate**
Wells et al. [31]	2015	serious	moderate	moderate	low	NI	moderate	moderate	**serious**
**Paediatric patients**									
Amado et al. [32]	2011	moderate	low	low	NI	moderate	moderate	low	**moderate**
Benner et al. [33]	2016	moderate	low	low	low	moderate	moderate	low	**moderate**
Cash et al. [34]	2014	serious	low	low	NI	low	serious	low	**serious**
Cohen et al. [35]	2016	moderate	low	low	low	NI	moderate	low	**moderate**
Corey et al. [36]	2008	serious	low	moderate	low	low	NI	low	**serious**
Dobrasz et al. [37]	2013	serious	low	moderate	low	NI	moderate	moderate	**serious**
Emerson et al. [38]	2018	serious	low	moderate	low	NI	Moderate	low	**serious**
Lamble et al. [39]	2015	moderate	low	low	moderate	NI	moderate	low	**moderate**
Lukes et al. [40]	2019	moderate	low	low	low	moderate	moderate	low	**moderate**
Monroe et al. [41]	2018	serious	low	low	NI	NI	moderate	serious	**serious**
Pakakasama et al. [42]	2011	serious	low	low	low	NI	moderate	moderate	**serious**
Salstrom et al. [16]	2015	moderate	low	low	low	low	moderate	moderate	**moderate**
Spencer et al. [43]	2017	moderate	low	moderate	Low	NI	moderate	low	**moderate**
Vanderway et al. [44]	2017	serious	low	low	low	NI	moderate	low	**moderate**
Vedi et al. [45]	2015	moderate	low	low	low	low	moderate	low	**moderate**
Volpe et al. [46]	2012	low	low	low	low	NI	moderate	low	**low**
Yoshida et al. [47]	2018	moderate	low	low	moderate	NI	moderate	low	**moderate**

Online Recourse 1, Table 2: Risk of bias assessment with ROBINS-I tool. *NI* no information; *pub*, published

*Corresponding author: C Koenig, Division of Pediatric Hematology/Oncology, Department of Pediatrics, Inselspital, Bern University Hospital, University of Bern, Bern, Switzerland; and Centre of Reviews and Dissemination, University of York, UK, christa.koenig@insel.ch

Professional (n=26)
• FN-Alert cards distributed to patients (7)<br>• Staff skills training (venous access) (6)<br>• Education of staff (24) (nurses, physicians, pharmacy)<br>• Educational updates/feedbacks (12)
**Organizational provider orientated (n=21)**
• Initiation of treatment by the nurse (3) Initiation of process by the nurse (1)<br>• Communication between professionals (10)<br>• Pharmacy assistance for antibiotic readiness (1)<br>• Sign and hold order/pre-arrival ordering (2)<br>• Delineate responsibilities for each team member (1)
**Organizational patient orientated (n=9)**
• Education of patients/parents (9)<br>• Instruction to put local anaesthetics for venous access (3)
**Organizational structural (n=28)**
• Implementation of: Checklist/Pathway/Protocol/Guidelines/Algorithm/Fast Pass Alert system (23)<br>• Revising/Setting up ED triage level for FN (8)<br>• Change triage system of FN patients (to private rooms at registration instead of waiting rooms / quicker access to infusion rooms) (5)<br>• Standardisation of FN definition (3)<br>• Target for TTA defined (8)<br>• Standardized order set (11)<br>• Standard dosing of antibiotics (4)<br>• New laboratory order set (faster release of ANC results / preliminary ANC test, rapid release of partial blood count) (3)<br>• Rapid ANC test (2)<br>• Intravenous access during blood sampling (1)<br>• Change in diagnostics (two blood cultures collected by single venepuncture, clinical evaluation after antibiotics) (2)<br>• Antibiotic administration before laboratory confirmation of neutropenia (11) (1x only for unstable patients)<br>• Close proximity to central line equipment (1)<br>• ED rooms prepared for potential septic patients (1)<br>• Altered admission process on computers (patient information can be entered before arrival of patient) (1)<br>• Definition of most appropriate antibiotic (7)<br>• Accurate antibiotics made available in ED/ICU (13)
**Other (n=1)**
• Alert letter to the general physician (1)

Online Resource 1, Table 3: Summary of interventions. *ANC*, absolute neutrophil count; *ED*, emergency department; *FN*, fever and neutropenia; *ICU*, intensive care unit; *TTA*, time to antibiotics

*Corresponding author: C Koenig, Division of Pediatric Hematology/Oncology, Department of Pediatrics, Inselspital, Bern University Hospital, University of Bern, Bern, Switzerland; and Centre of Reviews and Dissemination, University of York, UK, christa.koenig@insel.ch
